# Preventing Hepatitis B Virus Infection Among U.S. Military Personnel: Potential Impact of a 2-Dose Versus 3-Dose Vaccine on Medical Readiness

**DOI:** 10.1093/milmed/usac389

**Published:** 2022-12-16

**Authors:** Kimberly A Oelschlager, Michael S Termini, Catherine Stevenson

**Affiliations:** Navy Medicine Readiness and Training Unit Fallon, Fallon, NV 89406, USA; Navy Medicine Readiness and Training Command Beaufort, Beaufort, SC 29902, USA; Dynavax Technologies Corporation, Emeryville, CA 94608, USA

## Abstract

**Introduction:**

Hepatitis B, a major public health issue worldwide, has been associated with serious clinical outcomes. Military personnel are at particular risk for hepatitis B, such that hepatitis B vaccination is part of the accession process for new recruits. Although lost time costs and medical cost avoidance have been used by the U.S. Military to guide their decision-making protocols, this has not been applied to hepatitis B vaccination costs. Herein, a decision-analytic model is used to compare the effective vaccine protection rates and vaccine and operational costs of 2-dose versus 3-dose hepatitis B vaccine regimens in a population of recruits from the U.S. Marine Corps Recruit Depot, Parris Island.

**Methods:**

A decision-analytic model was developed to assess the expected levels of adherence, seroprotection, and vaccination and operational costs of a cohort of recruits vaccinated with either a 2-dose (HepB-CpG) vaccine for those eligible (scenario 1) or a 3-dose (HepB-Alum) vaccine (scenario 2). De-identified data from 23,004 recruits at the Marine Corps Recruit Depot, Parris Island, in 2018 and 2019 were used to provide real-world data on age distribution and vaccination status. Other inputs included published data on adherence for hepatitis B vaccines and seroprotection rates for HepB-CpG and HepB-Alum in relation to the number of doses received. Costs included direct medical costs of the hepatitis B vaccination and operational costs such as missed training time.

**Results:**

After receipt of two vaccine doses, 92% of recruits in scenario 1 (HepB-CpG group) were expected to be protected against hepatitis B within 1 month of receiving the second dose, compared with 24% of recruits in scenario 2 (HepB-Alum group), leaving 76% of Marine recruits unprotected if using HepB-Alum during the intervening 5-month period between doses 2 and 3. Over the study period, HepB-CpG was estimated to provide cost savings of $744,509 (17.3% cost reduction) compared with HepB-Alum, with the cost of missed training time being the most influential driver of the cost difference between the two vaccination schedules.

**Conclusions:**

Findings from this model suggest that vaccination with the 2-dose HepB-CpG vaccine may provide earlier and higher protection against hepatitis B compared with the 3-dose vaccine (HepB-Alum). A 2-dose vaccination strategy incorporated as part of individual medical readiness has the potential to not only increase protection but also confer economic savings among military recruits at risk for hepatitis B infection.

## INTRODUCTION

Infectious diseases occur worldwide, and military personnel are particularly vulnerable to infectious threats when deployed and posted overseas.^1^ Rates of hepatitis B infection are much higher in immigrants from holoendemic areas, a population important to U.S. Military recruitment goals, as well as in areas of military operations, where some estimates suggest that nearly 1 in 10 persons are infected with chronic hepatitis B.^2^ The overall incidence rates of acute and chronic hepatitis B infections among U.S. armed service members were both 10.0 per 100,000 person-years; however, it is likely that these reported rates are underestimates of the true burden of disease caused by chronic hepatitis B infection because such cases are generally asymptomatic.^3^ This has implications for individual medical readiness and force health protection. Since it was mandated in 2002, hepatitis B vaccination has been incorporated into the accession process for new recruits as a key component of medical readiness, with serologic testing required for evidence of immunity to the hepatitis B virus.^4,5^

Transmission of hepatitis B occurs through percutaneous, mucosal, or nonintact skin exposure to infected blood or body fluids; clinical manifestations of hepatitis B infection can range from asymptomatic infection to fulminant hepatitis that can result in death or hepatic failure.^6^ The U.S. Advisory Committee on Immunization Practices recommends hepatitis B vaccination for travelers to endemic regions.^6,7^ Furthermore, hepatitis B prevention and mitigation are part of predeployment planning.^8^ Hepatitis B in military personnel carries substantial human and fiscal costs, which may be underestimated because of cost shifting to Veterans’ Affairs and other health services. Hepatitis B is also a common cause of viral hepatitis among military personnel,^9^ who are likely to engage in activities that put them at risk of contact with the hepatitis B virus; consequences of infection vary but may take military personnel away from duties to receive acute medical care, whereas those who are chronic carriers of hepatitis B may experience long-term sequelae.^10^

First-generation aluminum adjuvanted hepatitis B vaccines (HepB-Alum; e.g., Engerix-B^®^ [referred to elsewhere as HBsAg-Eng or HepB-Eng], GlaxoSmithKline, Research Triangle Park, NC; Recombivax HB^®^, Merck Sharp & Dohme, Whitehouse Station, NJ) and combined hepatitis B and hepatitis A vaccines (e.g., Twinrix^®^ [HepA-HepB vaccine], GlaxoSmithKline), which are administered on a recommended 3-dose series over a 6-month interval, have been available for over 20 years.^11–13^ Available since 2017, HepB-CpG (HEPLISAV-B^®^, Dynavax Technologies, Emeryville, CA) is a 2-dose hepatitis B vaccine that can be administered over a 1-month period^14^ and has the potential to provide more immediate protection against the hepatitis B virus. Compared with hepatitis B vaccines given in a 3-dose series, HepB-CpG induces earlier and higher seroprotection rates (SPRs) among healthy adults 18 to 70 years of age, especially for adults who have historically had attenuated responses to 3-dose hepatitis B vaccines (e.g., smokers and males).^15–17^ Thus, HepB-CpG may represent an important tool to enable military personnel to achieve rapid protection against hepatitis B, including new recruits at risk of infection.

Because of the importance of medical readiness, U.S. DoD analyses have used lost time costs to improve decision-making, including assessments of training injuries, low back pain, and telemedicine care.^18–20^ The Army Public Health Center has also developed its own model of medical cost avoidance to contribute to informed decision-making that uses lost time costs as one of its inputs.^21^ However, to the best of our knowledge, this type of analysis has not been applied to hepatitis B vaccination costs. Therefore, we sought to compare the impact of using 2-dose versus 3-dose hepatitis B vaccine regimens in military recruits from the U.S. Marine Corps Recruit Depot, Parris Island, on both vaccine and operational costs, as well as on effective vaccine protection rates (eVPRs).

## METHODS

According to the Naval Medical Center Portsmouth Institutional Review Board (reference 935907), this project did not meet the definition of research in accordance with 32 CFR 219.102 and DoDI 3216.02. All numerical inputs and outputs presented here represent hypothetical retrospective modeling studies for illustrative purposes only.

### Model Description

**FIGURE 1.**
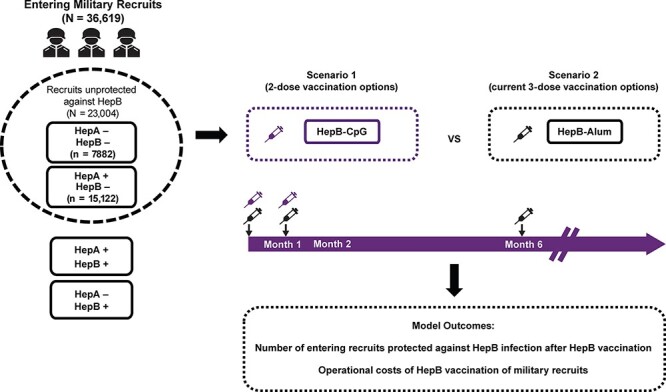
Model schematic. Entering recruits who were antibody negative for both hepatitis A and hepatitis B or only hepatitis B were considered unprotected against hepatitis B (*N* = 23,004). Unprotected Marine recruits received either a 2-dose (HepB-CpG; scenario 1) or a 3-dose (HepB-Alum; scenario 2) vaccine series based on their age and vaccination status. Note that because HepB-CpG is only approved for individuals ≥18 years of age, those <18 years of age received HepB-Alum. HepB = hepatitis B; HepA = hepatitis A.

A decision-analytic model was developed to assess operational costs and the anticipated level of serologic evidence of protection among newly accessioned Marine Corps recruits, following receipt of either a 2-dose (HepB-CpG) vaccine regimen (scenario 1) for those eligible (i.e., those ≥18 years of age) or a 3-dose (HepB-Alum) vaccine regimen (scenario 2; Fig. 1). Specifically, model outputs included operational costs derived from vaccination, missed training time, and administration, as well as the percentage of vaccinated military recruits with serologic evidence of immune protection against the hepatitis B virus (defined by HBsAg antibody level ≥10 mIU/mL).

The model used de-identified data from 23,004 new recruits at Parris Island in 2018 and 2019 who were either negative for hepatitis B and hepatitis A antibodies or negative for hepatitis B and positive for hepatitis A antibodies (Table S1). Upon model entry, Marine recruits who were hepatitis B antibody‒negative were assigned to groups based on their age and hepatitis B vaccination status (Tables S2 and S3).^22^ Entering recruits were assumed to receive either a 2- or 3-dose hepatitis B vaccination series (Table S2). The vaccination option for the 2-dose series was HepB-CpG; the vaccination option for the 3-dose series was HepB-Alum. Vaccination status upon entry was established with simple antibody titers, which were then used to determine whether a recruit would receive nothing or a hepatitis B vaccination. Recruits were assigned to receive pediatric or adult formulations of HepB-Alum based on age upon entry into the model (Table S2). Additionally, because the HepB-CpG vaccine is not licensed for individuals younger than 18 years,^14^ any recruits who were 17 years of age were assigned to receive HepB-Alum.

### Model Inputs: Operational Costs

The model used the cost of the vaccines as derived from the publicly available Federal Supply Schedule service prices at the time of vaccination (2018–2019), as well as the costs for missed training time and administration, and assumed 100% vaccination compliance to calculate the total vaccination cost (Table S4). It was assumed that costs for time out of field training and administration were the same for each individual vaccination encounter in each vaccine group. The missed training cost per clinic visit when receiving a vaccination in recruit training was assumed to be $140.00 (based on the Army Public Health Center’s Medical Cost Avoidance Model analysis of a 2-h per clinic visit during recruit training at Fort Sill).^21,23^ Missed training costs for recruit training on Parris Island were not included because clinic visits are part of the training schedule during the first 5 weeks post-accession (i.e., for doses 1 and 2); however, costs applied to HepB-Alum dose 3 when the recruits had advanced in their entry-level training continuum. The per-visit vaccine administration cost was $7.79, which was adjusted for inflation from an analysis of hepatitis B vaccination in a military recruit program.^24^

### Model Inputs: Compliance Rates

For the assessment of cost differences, the model assumed a 100% compliance rate. For assessment of seroprotection differences, the model assumed that 98% of recruits received all three doses of the 3-dose vaccine regimen based on the opinion of a military expert. The percentages of recruits who received only dose 1 or only doses 1 and 2 were 0.8% and 1.2%, respectively, and were based on a previously reported model.^25^

The model assumed that 99.2% of recruits received both doses of HepB-CpG, which was equal to the entirety of recruits having received two doses of the 3-dose vaccine regimen (Table S3). The percentage of patients receiving only one dose was then determined by subtracting the 2-dose compliance rate from 100%. The vaccination models assumed that HepB-CpG and the 3-dose vaccine would take 1 month after series completion to provide protection.

### Model Inputs: SPRs

SPRs for hepatitis B were derived from clinical trial data in adults (Table S3). The SPRs for HepB-CpG and HepB-Alum were sourced and pooled using data from three clinical trials in adults.^15–17^ For HepB-CpG dose 1, SPRs were pooled from week 4 of two clinical trials,^15,16^ and SPRs for dose 2 were pooled from week 24 of three clinical trials.^15–17^ For HepB-Alum, SPRs for doses 1 and 2 were pooled from week 4 or week 8 from two clinical trials,^15,16^ whereas dose 3 SPRs were pooled from week 28 from three clinical trials.^15–17^

### Model Estimation of Operational Cost Outcomes

For each vaccine, a total cost, including Federal Supply Schedule pricing and costs for missed training and administration time, was calculated as described earlier. Using the age and serologic status data from Parris Island, the number of Marine recruits in each group was multiplied by the total cost of that vaccine to determine the total cost of vaccination in each of the different scenarios.

### Model Estimation of Protection Outcomes

The potential real-world efficacy of immunization of new military recruits was measured by the eVPR. The eVPR estimates vaccine effectiveness by incorporating both vaccine efficacy (e.g., SPR when there is an accepted surrogate of protection) from clinical study data and real-world vaccine regimen adherence rates. The eVPR uses published vaccine compliance rates and SPRs to provide clinically relevant and value-driven analyses of adult hepatitis B vaccine effectiveness (Table S3; vaccine SPR [%] × recruits vaccinated by dose [%] = eVPR [%]).^26^ The eVPR for each dose of the 2- or 3-dose hepatitis B vaccines was determined by multiplying vaccine-specific compliance rates by the SPR for each dose.

The model calculated the number of Marine recruits protected against hepatitis B after each dose of either vaccine by summing the number of recruits protected by any previous dose(s) and the incremental number of recruits protected by that particular dose. For doses 1 and 2, calculations used a dose-specific eVPR to account for compliance rates that reflect the entirety of recruits receiving that dose. The number of recruits protected by only dose 1 was estimated by multiplying the eVPR for dose 1 by the number of unprotected recruits at month 0. To estimate the number of recruits protected by dose 2 only, the number of recruits unprotected at month 0 was multiplied by the eVPR for dose 2. To estimate the number of recruits protected after all three hepatitis B vaccine doses, the number of recruits unprotected at month 0 was multiplied by the eVPR for each dose (dose 1, 2, or 3) and summed. The number of recruits unprotected after each dose was determined by subtracting the number of recruits protected after that dose from the initial number of recruits entering the model.

### Analyses

#### Base case

Protection outcomes and operational costs were estimated under two alternative hepatitis B vaccination strategies: scenario 1, which is considered a 2-dose (HepB-CpG) vaccine series, and scenario 2, which is considered a 3-dose (HepB-Alum) vaccine series. In this analysis, 23,004 Marine recruits 17 years of age and older were assigned to either vaccine group following the age breakdown shown in Table S3. Protection outcomes were measured 1 month after each dose of both vaccines, and operational costs for each strategy were projected by estimating the total spend per strategy and spend per recruit.

#### Sensitivity analyses

One-way sensitivity analyses were conducted to assess the effect of input parameter uncertainty on operational costs. In this analysis, key model parameters (missed training time cost and administration cost) were altered one at a time by assuming 15% higher or lower values than the base case inputs (Table S5). The total spend cost difference following vaccination with either HepB-CpG or HepB-Alum was evaluated.

A scenario analysis was also conducted to evaluate the influence of compliance rate on the number of military recruits protected after completing HepB-CpG or HepB-Alum vaccination, assuming vaccine compliance rates based on a recent real-world adherence study in the general population (Table S6).^27^

## RESULTS

### Protection Rates After Hepatitis B Vaccination Among Military Recruits

**FIGURE 2.**
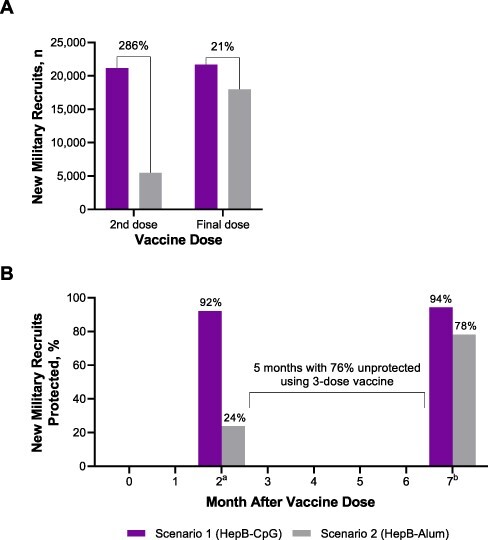
Estimated (A) number protected and (B) percentage protected among Marine recruits after vaccination with either HepB-CpG or HepB-Alum. ^a^After two doses. ^b^After final dose.

The model included 23,004 Marine recruits entering Parris Island who were unprotected against hepatitis B, of whom 4.1%, 48.1%, 23.0%, and 24.7% were 17, 18, 19, and ≥20 years of age, respectively (Table S3). The estimated number of recruits protected after receiving each dose of either HepB-CpG or HepB-Alum is shown in Table S7. After two doses of HepB-CpG, an anticipated 21,185 recruits would be protected against hepatitis B, which corresponds to 286% more recruits protected with two doses of HepB-CpG than with two doses of HepB-Alum (Fig. 2A). Upon series completion, HepB-CpG was estimated to protect an additional 21% of recruits compared with HepB-Alum. The model estimated that 76% of Marine recruits receiving HepB-Alum would be unprotected against hepatitis B in the 5 months between receipt of the second and final doses (i.e., between months 2 and 7; Fig. 2B).

### Operational Costs of Hepatitis B Vaccination of Military Recruits

**FIGURE 3.**
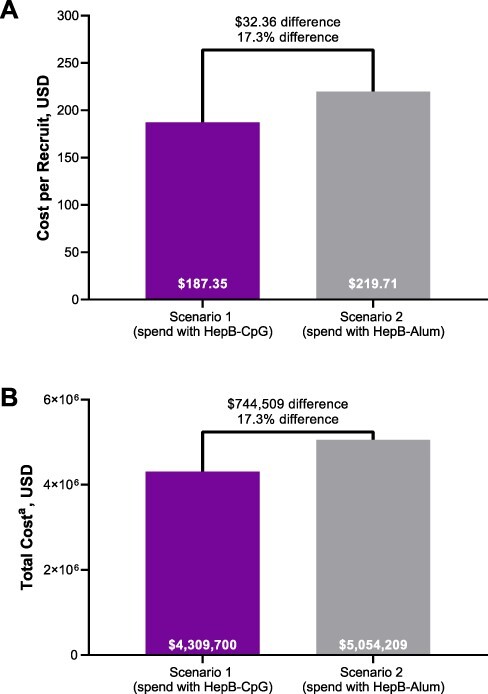
Costs (A) per recruit and (B) in total among Marine recruits after vaccination with either HepB-CpG or HepB-Alum. ^a^Based on 23,004 recruits. USD = U.S. dollars.

The operational costs of vaccinating Marine recruits with either HepB-CpG or HepB-Alum are summarized in Figure 3. Assuming missed training time and administration costs of $140 and $8, respectively, the cost per recruit was $187.35 with HepB-CpG and $219.71 with HepB-Alum. Based on 23,004 new recruits being unprotected against hepatitis B, the total costs for HepB-CpG and HepB-Alum were $4,309,700 and $5,054,209, respectively. This equates to a 17.3% cost decrease and a total cost savings of $744,509 with HepB-CpG versus HepB-Alum.

### Sensitivity Analyses

**FIGURE 4.**
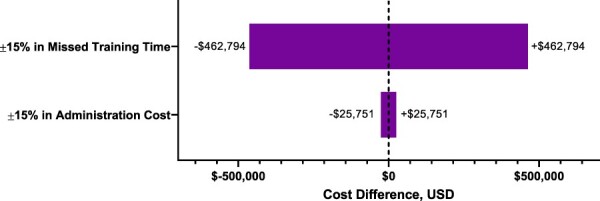
One-way ±15% sensitivity analysis on influential variables. USD = U.S. dollars.

The one-way sensitivity analysis between HepB-CpG and HepB-Alum determined that the incremental cost was most sensitive to the missed training time cost (Fig. 4). For the scenario analysis assuming lower hepatitis B vaccine compliance rates,^27^ there was a 91.2% difference in the number of protected recruits from the base case scenario (*n*, base case: 3702; sensitivity analysis: 7077).

## DISCUSSION

Chronic infection with hepatitis B is a major public health issue worldwide^28^ and can lead to serious clinical outcomes, including liver cirrhosis and failure, cancer, and premature death.^6^ Persons with chronic hepatitis B infection are the main reservoirs of viral transmission, with approximately 248 million individuals with chronic infection worldwide and an estimated 840,000 persons in the United States.^28^ Military personnel are at particular risk of hepatitis B infection because of deployment to countries in which hepatitis is endemic^8^ and their likely engagement in activities that put them at increased risk of contact with the hepatitis B virus.^10^ Furthermore, the associated immediate and long-term costs of hepatitis B infection among military personnel provide an important rationale for vaccination against hepatitis B as mandated for new U.S. military recruits as a key component of medical readiness.^5,6,10^

Hepatitis B virus vaccines are available in the United States and are administered on either a 2- or 3-dose schedule^11,12,14,29^; however, the specific regimen recommended for U.S. Military recruits is not specified. Compared with 3-dose vaccines (0-, 1-, and 6-month schedule), higher and earlier seroprotection in adults is observed with the use of HepB-CpG,^15–17^ administered as two doses, 1 month apart.^14^ Therefore, HepB-CpG could be an important tool to enable rapid protection against hepatitis B, particularly among new military recruits at risk of infection. However, an important consideration in implementing a vaccination program is the assessment of the likely eVPR and cost outcomes of such a strategy. Because of the importance placed on individual medical readiness, U.S. DoD analyses have used lost time costs and medical cost avoidance in their decision-making protocols,^18–21^ but this approach has not been applied to vaccination costs. Therefore, we used a hypothetical model to compare eVPR as well as vaccine and operational costs of 2-dose versus 3-dose hepatitis B vaccine regimens in a population of new Marine recruits.

Findings from our study showed that implementing a 2-dose hepatitis B vaccine regimen for Marine recruits results in a higher number of personnel achieving protection versus a 3-dose vaccine regimen. Higher rates of protection with HepB-CpG versus a 3-dose vaccine are an important finding because a recent study of U.S. military veterans showed higher hepatitis B exposure rates than the general population, with 14% of veterans exposed to hepatitis B and higher exposure rates among veterans exposed to blood in combat or with HIV or cirrhosis.^30^

Our study also found considerable monetary savings in military operational costs when utilizing a 2-dose versus a 3-dose hepatitis B vaccine regimen for incoming recruits, with HepB-CpG estimated to provide a 17.3% reduction in spending on operational costs compared with HepB-Alum over the study period. The cost of missed training time was the most influential driver of the cost difference between the two vaccination schedules. Our results are consistent with previous modeling studies from the United States that showed that HepB-CpG was a cost-effective interventional strategy among multiple at-risk populations,^31–33^ including those at risk because of occupational and environmental exposure.^31^ These findings are important, given that downstream medical costs associated with lack of protection against hepatitis B remain substantial, with previous analyses finding annual costs of $6840 and $35,976 for compensated and decompensated cirrhosis, respectively, and $31,916 for hepatocellular carcinoma resulting from occupational and international exposure.^31^

In addition to the favorable seroprotection and operational cost impacts, the use of a 2-dose vaccine for protecting new recruits against hepatitis B may also be logistically simpler. Following the formal recruit training period on Parris Island, the Marines will advance in their entry-level training continuum. With a 3-dose vaccine, the third dose at 6 months would need to be administered after this advancement. However, operational commitments and rigorous schedules often preclude timely administration of the third dose, raising the potential for vaccination remaining incomplete by 6 months. Furthermore, there will be additional cost incurred at the training continuum locations (e.g., School of Infantry or Marine Combat Training).

Potential study limitations include that our analysis assumed that missed training costs were not applicable during recruit training on Parris Island and thus were not applicable for doses 1 and 2 of either vaccine regimen; however, even if missed training costs were considered for these doses, the actual difference would remain constant. In addition, our model considered a 98% compliance rate for series completion of the 3-dose vaccine regimen, which would occur when the Marines are in entry-level training continuum locations, but this is based on expert opinion of high compliance in military recruits. Actual compliance rates for mandated vaccinations in Marines may be lower. A sensitivity analysis assuming lower compliance rates demonstrated that HepB-CpG would similarly offer higher protection than a 3-dose vaccine. Relatedly, because 1-dose and 2-dose hepatitis B vaccine compliance rates among new military recruits were not available in the literature, these rates were instead estimated by incorporating compliance rates among the general population of the United States. Accordingly, these compliance rates may not reflect actual rates among new military recruits.

## CONCLUSIONS

Vaccinating incoming U.S. Military recruits with the 2-dose HepB-CpG vaccine has the potential to provide earlier and higher protection against hepatitis B compared with 3-dose vaccines. When incorporated as part of individual medical readiness, the 2-dose vaccination strategy could increase protection and confer economic savings among the population of military recruits who are at risk of hepatitis B infection.

## Supplementary Material

usac389_Supp

## Data Availability

The datasets generated during and/or analyzed during the current study are available from the corresponding author on reasonable request.
