# Supplementary material for: Preventing Hepatitis B Virus Infection Among U.S. Military Personnel: Potential Impact of a 2-Dose Versus 3-Dose Vaccine on Medical Readiness
Source: Mil Med. 2022 Dec 16;188(7-8):e2067–73. doi: 10.1093/milmed/usac389 (PMC10362997; doi:10.1093/milmed/usac389)
Supplement: usac389_Supp [file usac389_supp.zip › (Oleschlager) 2v3 Dose Military Recruits_Revised_Supplement.docx]

# Supplemental Materials

# Table S1. Summary of Hepatitis B Status of Marine Corps Recruit Depot, Parris Island Recruits Included in Model

|  | **Number of Recruits** |
| --- | --- |
| Total recruits | 36,619 |
| Hepatitis B antibody negative | 23,004 (63% of total population) |
| Hepatitis B negative and Hepatitis A positive | 15,122 (66% of hepatitis B-negative population) |
| Hepatitis B negative and Hepatitis A negative | 7,882 (34% of hepatitis B-negative population) |
| Hepatitis B antibody positive | 13,615 (37% of total population) |

# Table S2. Vaccine Options Stratified by Age Group and Vaccination Status^9,12^

| **Vaccine (Dose; Schedule)** | **Age Group** | | | | |  | **Vaccination Status** | |
| --- | --- | --- | --- | --- | --- | --- | --- | --- |
|  | **17 Years** | **18 Years** | **19 Years** | **20 Years** | **≥21 Years** |  | **Hepatitis B Antibody Negative** | **Hepatitis B and A Antibody Negative** |
| **2-dose vaccine** |  |  |  |  |  |  |  |  |
| HepB-CpG* (0.5 mL; 0, 1 month) |  |  |  |  |  |  |  |  |
| **3-dose vaccine** |  |  |  |  |  |  |  |  |
| HepB-Alum adult^†^ (1 mL; 0, 1, 6 months) |  |  |  |  |  |  |  |  |
| HepB-Alum pediatric^‡^ (0.5 mL; 0, 1, 6 months) |  |  |  |  |  |  |  |  |

*18 years and older.
^†^20 years and older.
^‡^Up to 19 years of age.

**Table S3. Model Inputs: Population Age, SPRs, Compliance Rates, and Effective VPRs**

|  | **Hepatitis B Vaccine Option** | |
| --- | --- | --- |
|  | **Scenario 1**  **(2-Dose Vaccine)** | **Scenario 2**  **(3-Dose Vaccine)** |
|  | **HepB-CpG** | **HepB-Alum** |
| Recruit age, % |  |  |
| 17 years | 4.1 | 4.1 |
| 18 years | 48.1 | 48.1 |
| 19 years | 23.0 | 23.0 |
| ≥20 years | 24.7 | 24.7 |
| Vaccine SPR, %^*^ |  |  |
| Dose 1 | 22.0 | 4.2 |
| Dose 2 | 95.7 | 24.0 |
| Dose 3 | N/A | 79.5 |
| Recruits vaccinated by dose, %^†^ |  |  |
| Dose 1 | 0.8 | 0.8 |
| Doses 1 and 2 | 99.2 | 1.2 |
| Doses 1, 2, and 3 | N/A | 98.0 |
| Effective VPR, %^‡^ |  |  |
| Dose 1 | 0.2 | 0.03 |
| Dose 2 | 94.9 | 0.3 |
| Dose 3 | N/A | 77.9 |
| Total effective VPR | 95.1 | 78.2 |

N/A=not applicable; SPR=seroprotection rate; VPR=vaccine protection rate.

^*^SPRs for HepB-Alum and HepB-CpG were derived from 3 pivotal clinical trials.^15-17^

^†^Compliance rates were derived from the assumption that 98% of recruits receive all doses of the 3-dose vaccine regimen based on the opinion of a military expert.

^‡^The per-dose effective VPR was determined by multiplying vaccine-specific compliance rates by the SPR for that dose. Total effective VPR reflects the sum of per-dose effective VPRs.

# Table S4. Model Inputs: Operational Costs

|  | **Operational Cost, $** |
| --- | --- |
| Missed training time* | 140.00 |
| Administration^†^ | 8.00 |
| Hepatitis B Vaccine (cost per dose)^‡^ |  |
| HepB-CpG | 85.65 |
| HepB-Alum pediatric | 13.11 |
| HepB-Alum adult | 36.34 |

*Derived from an analysis of cost for every hour ($70/h) of training missed in Army recruit training while in clinic at Fort Sill and adjusted for inflation. Missed training time was assumed to only be applicable following Recruit Training on Parris Island and thus was not applied for doses 1 or 2 of either vaccine.
^†^Vaccination administration costs were based on an analysis of a school-based hepatitis B vaccination program^24^ and adjusted for inflation. Administration costs were assumed applicable for all doses.

^‡^Derived from the US Federal Supply Schedule service award pricing.^34^

# Table S5. Inputs for One-Way ±15% Sensitivity Analysis

| **Factor Changed** | **Cost, $** | | |
| --- | --- | --- | --- |
|  | **Base Case** | **−15% Number** | **+15% Number** |
| Missed training time | 140.00 | 119.00 | 161.00 |
| Administration | 7.79 | 6.62 | 8.96 |

# Table S6. Hepatitis B Virus Vaccine Compliance Rate Inputs for Scenario Analysis

|  | **Recruits Receiving Dose, %** |
| --- | --- |
| Scenario 1 (HepB-CpG) |  |
| Dose 1 | 37.4 |
| Dose 2 | 62.6 |
| Scenario 2 (HepB-Alum) |  |
| Dose 1 | 37.3 |
| Dose 2 | 27.8 |
| Dose 3 | 34.9 |

# Table S7. Estimated Number of Military Recruits Protected With HepB-CpG Versus HepB-Alum

|  | **Scenario 1**  **HepB-CpG**  (N=23,004*) | **Scenario 2**  **HepB-Alum**  (N=23,004) |
| --- | --- | --- |
| After dose 1, n (%) | 4888 (21.2) | 966 (4.2) |
| After dose 2, n (%) | 21,185 (92.1) | 5485 (23.8) |
| Increase in recruits protected with HepB-CpG, % | 286 |  |
| After all doses, n (%) | 21,698 (94.3) | 17,996 (78.2) |
| Increase in recruits protected with HepB-CpG, % | 21 |  |

*Because HepB-CpG is not approved for 17-year-olds, recruits in this age bracket (n = 950) were instead administered HepB-Alum.
